# The Comparison of the Outcomes between Primary PCI, Fibrinolysis, and No Reperfusion in Patients ≥ 75 Years Old with ST-Segment Elevation Myocardial Infarction: Results from the Chinese Acute Myocardial Infarction (CAMI) Registry

**DOI:** 10.1371/journal.pone.0165672

**Published:** 2016-11-03

**Authors:** He Peiyuan, Yang Jingang, Xu Haiyan, Gao Xiaojin, Xian Ying, Wu Yuan, Li Wei, Wang Yang, Tang Xinran, Yan Ruohua, Jin Chen, Song Lei, Zhang Xuan, Fu Rui, Ye Yunqing, Dong Qiuting, Sun Hui, Yan Xinxin, Gao Runlin, Yang Yuejin, on behalf of the CAMI Registry study group

**Affiliations:** 1 Department of Cardiology, Cardiovascular Institute and Fuwai Hospital, National Center for Cardiovascular Diseases, Chinese Academy of Medical Sciences and Peking Union Medical College, Beijing, 100037, China; 2 Duke Clinical Research Institute, Duke University Medical Center, Durham, NC, United States of America; 3 Medical Research & Biometrics Center, Cardiovascular Institute and Fuwai Hospital, National Center for Cardiovascular Diseases, Chinese Academy of Medical Sciences and Peking Union Medical College, Beijing, 100037, China; University of Louisville, UNITED STATES

## Abstract

**Background:**

Only a few randomized trials have analyzed the clinical outcomes of elderly ST-segment elevation myocardial infarction (STEMI) patients (≥ 75 years old). Therefore, the best reperfusion strategy has not been well established. An observational study focused on clinical outcomes was performed in this population.

**Methods:**

Based on the national registry on STEMI patients, the in-hospital outcomes of elderly patients with different reperfusion strategies were compared. The primary endpoint was defined as death. Secondary endpoints included recurrent myocardial infarction, ischemia driven revascularization, myocardial infarction related complications, and major bleeding. Multivariable regression analysis was performed to adjust for the baseline disparities between the groups.

**Results:**

Patients who had primary percutaneous coronary intervention (PCI) or fibrinolysis were relatively younger. They came to hospital earlier, and had lower risk of death compared with patients who had no reperfusion. The guideline recommended medications were more frequently used in patients with primary PCI during the hospitalization and at discharge. The rates of death were 7.7%, 15.0%, and 19.9% respectively, with primary PCI, fibrinolysis, and no reperfusion (P < 0.001). Patients having primary PCI also had lower rates of heart failure, mechanical complications, and cardiac arrest compared with fibrinolysis and no reperfusion (P < 0.05). The rates of hemorrhage stroke (0.3%, 0.6%, and 0.1%) and other major bleeding (3.0%, 5.0%, and 3.1%) were similar in the primary PCI, fibrinolysis, and no reperfusion group (P > 0.05). In the multivariable regression analysis, primary PCI outweighs no reperfusion in predicting the in-hospital death in patients ≥ 75 years old. However, fibrinolysis does not.

**Conclusions:**

Early reperfusion, especially primary PCI was safe and effective with absolute reduction of mortality compared with no reperfusion. However, certain randomized trials were encouraged to support the conclusion.

## Introduction

The global number of people aged > 65 years is estimated to reach 540 million in 2015 and about 1 billion in 2025.[1]Older people frequently have higher rates of comorbidities and cardiovascular risk factors, which place them at higher risk of contracting coronary artery disease than younger individuals.[2–4] ST-segment elevation myocardial infarction (STEMI) is the most serious condition of all coronary artery disease presentations. Previous studies have suggested that STEMI patients of older age are at higher risk of death, bleeding, and complications regardless of treatment.[5]However, with the development and introduction of new antithrombotic drugs, technologies, and devices, the outcomes of older patients have improved.[6]Immediate reperfusion of the infarct artery is the best treatment for STEMI patients. However, the reperfusion strategy, by either fibrinolysis or primary percutaneous coronary intervention(PCI) in older patients, has seldom been compared. Elderly patients are at a higher risk of bleeding,[7,8]which is great concern for doctors in selecting the reperfusion strategy, and the outcomes in older patients without reperfusion were also unknown. Although the effectiveness and safety of primary PCI among younger patients (<75 years old) has been proven through randomized trials,[9,10]elderly patients (≥ 75 years old) are usually excluded.[11]Some randomized trials designed in evaluating the outcomes of PCI and fibrinolysis in older people have to terminate earlier, because of the slow progress in recruiting patients. Current guidelines showed no specific recommendations for older STEMI patients in regard to the reperfusion strategy because of the deficiency of evidence. Given the limited knowledge on the effect of different treatment strategies and the corresponding outcomes of elderly patients, the in-hospital outcomes of elderly patients (age ≥ 75) were compared between primary PCI, fibrinolysis, and no reperfusion, from a “real-world” contemporary era using the database of a national registry study.

## Materials and Methods

### Study design

The Chinese Acute Myocardial Infarction (CAMI) Registry is a national registry study (NCT01874691). The study started enrolling patients in 2013. Currently, more than 100 hospitals located around China have participated. The CAMI registry was designed to reflect an unbiased and representative sample for the treatment and outcomes of patients with acute myocardial infarction (MI) in China. Full details of the rationale and methodology of the CAMI registry study have been depicted elsewhere.[12]

### Data collection

From January 1, 2013 to Sep 30, 2014, 26,592 patients were enrolled. Among them, 19,241 patients were diagnosed with STEMI. 3151 patients of them were ≥ 75 years old, and 69 patients were excluded because the reperfusion information was missing. Finally 3,082 patients were analyzed, and among them, 1000 patients had primary PCI, 160 patients had fibrinolysis, and 1922 patients had no reperfusion. Patient data at each site was input into computer using standardized electronic case report form, and transferred to the database center through the internet by trained research doctors. The case report form included the patients’ demographic characteristics, presenting symptoms, medical histories, in-hospital treatments, major outcomes, and discharged medications. The protocol of this study was approved by the Ethics Committee of Fuwai Hospital and the local institution. Written informed consents were obtained on patients’ admission. Patient information was de-identified prior to analysis.

### Definitions

The primary endpoint for the analysis was defined as death. The secondary endpoint was defined as recurrent MI, ischemia-driven revascularization (IDR), cerebral events (ischemic or hemorrhage stroke), major bleeding (excluding hemorrhage stroke), MI related complications, which included heart failure, mechanical complications, ventricular tachycardia/ventricular fibrillation, and cardiac arrest. Recurrent MI was defined by meeting at least two of the following criteria: unrelieved onset of chest pain longer than 20 min; elevation of myocardial enzymes (troponin T, troponin I, or creatine kinase-MB) that are more than three times the upper reference limit; and new changes in the ST-segment or Q waves in electrocardiograms, indicating new muscle injuries. IDR was defined as an emergent intervention induced by the ischemia of a previously treated vessel or new thrombosis. Mechanical complications included perforation of ventricular septum, rupture of papillary muscles, rupture of free ventricular wall, and acute and subacute cardiac ruptures. Major bleeding (excluding hemorrhage stroke) was defined as bleeding meeting one of the following criteria: drop of hemoglobin more than 3 g/L, needing transfusion, needing surgical intervention, or extra hospital care (simple compression was not included). We used an effective risk evaluation system, the Global Registry of Acute Coronary Events (GRACE) risk score to evaluate patients’ death risk on admission. An accumulated score <109 points was considered as low-risk, between 109 to 140 points as moderate high-risk, and >140 points as very high-risk.[13]

### Statistics

Patients were initially stratified into 3 groups: patients had primary PCI, patients had fibrinolysis, and patients had no reperfusion. The baseline characteristics, medications administrated, and major endpoints were compared between the 3 groups. Continuous variables were expressed as mean ± standard deviation and compared using variance analysis. Abnormally distributed variables were shown as medians with quartile range and compared using Wilcoxon rank sum test. Categorical variables were expressed as frequencies with percentages and compared using Chi-square test or Fisher’s exact test when applicable. To balance the disparities between the groups, a multivariate logistic model was used to adjust for the potential confounding. In this model, univariate analysis was primarily performed to test the associations between each baseline characteristics and the primary endpoint. The variables with significance at P < 0.1 were included in the multivariate model. Finally variables that included in the model were as follows: GRACE score stratification (categorized as low-risk, moderate high-risk, and very high-risk), sex, hospital level, prior stroke, prior heart failure, current smoking, left ventricular ejection fraction, diastolic pressure, use of intra-aortic balloon pump, in-hospital medication of aspirin, adenosine diphosphate inhibitor, anticoagulation agent, statin, βblocker, angiotensin-converting enzyme inhibitor/ angiotensin receptor blocker (ACEI/ARB), nitrate. For all analyses, two-tailed P < 0.05 was considered statistically significant, and all analyses were performed using SAS statistical package, version 10.2 (Cary, NC).

## Results

Patient baseline characters are shown in Table 1. Patients who had no reperfusion were relatively older (P < 0.001). Men were more likely to have primary PCI (64.3%) or fibrinolysis (62.5%) compared with women. The average time from symptom onset to treatment was different in three groups. Patients who came to hospital earlier (< 6 h) had higher chance obtaining fibrinolysis or primary PCI, while those coming late (> 12 h) were more frequently treated with no reperfusion. The level of the hospital also affected the choice of the revascularization strategy. More than 95% of the primary PCI was performed in provincial or municipal hospitals, while around 90% fibrinolyisis were performed in municipal ortertiary hospitals. Most of patients' medical histories were similar between three groups, except that patients who had primary PCI also had fewer prior strokes (P = 0.005). The cardiovascular risk factors of the patients were comparable between the groups, except the rates of dislipidemia and current smoking status were slightly different. The mean value of left ventricular ejection fraction was different (P < 0.001). However, the difference was not great. When we used GRACE score to evaluate patient’s risk, we found patients who had primary PCI or fibrinolysis were of relatively lower risk compared with patients who had no reperfusion. We also observed that the primary PCI group had more Killip I patients, and less Killip IV patients than the other groups.

**Table 1:** Baseline characters.

Variables	Fibrinolysis (N = 160)	Primary PCI (N = 1000)	No reperfusion (N = 1922)	P value
Age (year)	78.79 ±7.12	79.59 ±4.04	80.59 ±4.57	<0.001
Male	100 (62.5%)	643 (64.3%)	1086 (56.5%)	<0.001
Time from symptom onset to treatment				<0.001
<3 h	51 (31.9%)	278 (27.8%)	246 (12.8%)	
3–6 h	61 (38.1%)	372 (37.2%)	344 (17.9%)	
6–12 h	19 (11.9%)	229 (22.9%)	264 (13.7%)	
12–24 h	11 (6.9%)	56 (5.6%)	285 (14.8%)	
1–7 d	18 (11.3%)	65 (6.5%)	783 (40.7%)	
Hospital level				<0.001
Province level	18 (11.3%)	449 (44.9%)	404 (21.0%)	
Municipal level	81 (50.6%)	509 (50.9%)	1082 (56.3%)	
Tertiary level	61 (38.1%)	42 (4.2%)	436 (22.7%)	
Medical history				
Prior MI	14 (8.8%)	54 (5.4%)	135 (7.0%)	0.127
Prior CABG	1 (0.6%)	3 (0.3%)	5 (0.3%)	0.767
Prior PCI	6 (3.8%)	41 (4.1%)	57 (3.0%)	0.272
Prior stroke	26 (16.3%)	98 (9.8%)	258 (13.4%)	0.005
Prior HF	6 (3.8%)	21 (2.1%)	70 (3.6%)	0.057
PAD	0 (0.0%)	7 (0.7%)	14 (0.7%)	0.324
Prior bleeding	1 (0.6%)	18 (1.8%)	50 (2.6%)	0.096
CKD	1 (0.6%)	11 (1.1%)	30 (1.6%)	0.387
Risk factors				
Diabetes	25 (15.6%)	170 (17.0%)	326 (17.0%)	0.904
Hypertension	95 (59.4%)	563 (56.3%)	1063 (55.3%)	0.570
Dislipidemia	6 (3.8%)	66 (6.6%)	44 (2.3%)	<0.001
Currently Smoking	66 (41.3%)	389 (38.9%)	664 (34.5%)	0.028
Admission presentation				
LVEF (%)	52 [48.5, 55]	52 [48, 58]	52 [47, 54]	<0.001
Heart rate (bpm)	73 [62.5 84.5]	74 [63, 85]	79 [67, 92]	<0.001
SBP (mmHg)	124.5 [110, 142.5]	125 [110, 142]	127 [110, 144]	0.556
DBP (mmHg)	75 [65, 88.5]	75 [64, 83]	75 [65, 85]	0.689
Killip classification				<0.001
I	100 (62.5%)	718 (71.8%)	1062 (55.3%)	
II	33 (20.6%)	214 (21.4%)	513 (26.7%)	
III	15 (9.4%)	29 (2.9%)	204 (10.6%)	
IV	12 (7.5%)	39 (3.9%)	143 (7.4%)	
GRACE score				<0.001
Very high risk	107 (66.9%)	659 (65.9%)	1456 (75.8%)	
Moderate high risk	50 (31.3%)	333 (33.3%)	452 (23.5%)	
Low risk	3 (1.9%)	8 (0.8%)	14 (0.7%)	

MI, myocardial infarction; CABG, Coronary artery bypass graft; PCI, percutaneous coronary intervention; HF, heart failure; PAD, Peripheral artery disease; CKD, chronic kidney disease; LVEF, left ventricular ejection fraction; SBP, systolic blood pressure; DBP, diastolic blood pressure;

The medication administrated during hospitalization and at discharge is shown in Table 2. Patients having primary PCI were more likely to receive anti-platelet agents including aspirin (98.2%), Adenosine diphosphate inhibitor (98.1%), GP IIb/IIIa inhibitors (46.9%) than patients having fibrinolysis and no reperfusion (P < 0.05). On the contrary, patients with no reperfusion were more likely to receive anti-coagulation agents (P = 0.005) than the other groups. In-hospital statin, β-blocker, ACEI/ARB were more frequently used in the primary PCI group (P < 0.05). However, nitrate was less used in the primary PCI group than in the fibrinolysis and no reperfusion group (58.4%, 76.9%, and 76.6% respectively, P < 0.001). Similar trends were detected in the medication at patients’ discharge.

**Table 2:** Medication.

Variables	Fibrinolysis (N = 160)	Primary PCI (N = 1000)	No reperfusion (N = 1922)	P value
**In-hospital**				
Aspirin	151 (94.4%)	982 (98.2%)	1770 (92.1%)	<0.001
ADP inhibitor	150 (93.8%)	981 (98.1%)	1775 (92.4%)	<0.001
GP IIb/IIIa inhibitor	11 (6.9%)	469 (46.9%)	186 (9.7%)	<0.001
Anticoagualtion drug	152 (95.0%)	951 (95.1%)	1868 (97.2%)	0.014
LWMH	147 (91.9%)	877 (87.7%)	1800 (93.7%)	<0.001
Fondaparinux	5 (3.1%)	74 (7.4%)	68 (3.5%)	<0.001
Statin	154 (96.3%)	980 (98.0%)	1826 (95.0%)	<0.001
β-blocker	90 (56.3%)	669 (66.9%)	1087 (56.6%)	<0.001
ACEI/ARB	88 (55.0%)	584 (58.4%)	986 (51.3%)	0.001
Nitrate	123 (76.9%)	584 (58.4%)	1472 (76.6%)	<0.001
**At discharge**				
Aspirin	143 (89.4%)	904 (90.4%)	1635 (85.1%)	<0.001
ADP inhibitor	138 (86.3%)	896 (89.6%)	1592 (82.8%)	<0.001
Statin	144 (90.0%)	902 (90.2%)	1668 (86.8%)	0.018
β-blocker	107 (66.9%)	634 (63.4%)	1147 (59.7%)	0.047
ACEI/ARB	78 (48.8%)	468 (46.8%)	762 (39.6%)	<0.001
Nitrate	114 (71.3%)	471 (47.1%)	1207 (62.8%)	<0.001

ADP, Adenosine diphosphate; GP Glyco-protein; LWMH, low weight molecule heparin; ACEI/ARB, angiotensin-converting enzyme inhibitor/ angiotensin receptor blocker

The clinical outcomes are shown in Table 3. The mortality rate in the primary PCI group was significantly lower than that in the fibrinolysis and no reperfusion group (7.7%, 15.0%, and 19.9% respectively, P < 0.001). Meanwhile, the rates of cardiac death (7.3%, 14.4% and 18.6% respectively, P < 0.001) and non-cardiac death (0.6%, 0.6%, and 1.4% respectively, P = 0.028) were also lower in the primary PCI group than that in the fibrinolysis and no reperfusion group. Recurrent MI was similar between the groups (P = 0.412). However, IDR was more common in patients having primary PCI (P = 0.007). MI related complications, which included heart failure, mechanical complications, and cardiac arrest occurred less in the primary PCI group(P < 0.05). On the contrary, the rates of ventricular tachycardia/ventricular fibrillation, cerebral events, hemorrhage stroke, and major bleeding (excluding hemorrhage stroke) were similar between the primary PCI, fibrinolysis, and no reperfusion groups (P < 0.05). Moreover, we found patients in the primary PCI group were more likely to obtain intra-aortic balloon pump support.

**Table 3:** Clinical outcomes with different reperfusion strategies.

Variables	Fibrinolysis (N = 160)	Primary PCI (N = 1000)	No reperfusion (N = 1922)	P value
Death	24 (15.0%)	77 (7.7%)	383 (19.9%)	<0.001
Cardiac death	23 (14.4%)	73 (7.3%)	357 (18.6%)	<0.001
Non-cardiac death	1 (0.6%)	4 (0.6%)	26 (1.4%)	0.028
Recurrent MI	3 (1.9%)	7 (0.7%)	16 (0.8%)	0.412
Ischemia driven revascularization	0 (0.0%)	9 (0.9%)	3 (0.2%)	0.007
MI-related complications				
Heart failure	46 (28.8%)	177 (17.7%)	636 (33.1%)	<0.001
Mechanical complications	3 (1.9%)	5 (0.5%)	35 (1.8%)	0.006
VT/VF	7 (7.5%)	32 (5.2%)	65 (5.1%)	0.636
Cardiac arrest	10 (6.3%)	36 (3.6%)	192 (10.0%)	<0.001
Cerebral events	3 (1.9%)	17 (1.7%)	29 (1.5%)	0.889
Hemorrhage stroke	1 (0.6%)	3 (0.3%)	1 (0.1%)	0.094
Other major bleeding	8 (5.0%)	30 (3.0%)	59 (3.1%)	0.439
Transfusion	1 (0.6%)	9 (0.9%)	11 (0.6%)	0.605
IABP support	1 (0.6%)	73 (7.3%)	31 (1.6%)	<0.001
Pulmonary embolism	0 (0.0%)	0 (0.0%)	3 (0.2%)	0.244
ICU or CCU stay (d)	4 (1, 7)	3 (2, 6)	3 (0, 7)	<0.001
Normal ward stay (d)	6 (0, 11)	5 (3, 8)	5 (1, 10)	0.606

MI, myocardial infarction; VT/VF, ventricular tachycardia/ventricular fibrillation; IABP, intra-aortic balloon pump; ICU, intensive care unit; CCU, coronary care unit.

So as to balance the baseline disparities and the selection bias, we used logistic model to adjust for the potential confounding. The adjusted outcomes are shown in Table 4. We found that the mortality rate was still significantly lower in the primary PCI group than in the no reperfusion group after adjustment(OR: 0.424, 95% CI:0.316, 0.569, P < 0.001), and the difference reached board significance when primary PCI was compared with fibrinolysis (OR: 0.592, 95% CI:0.343, 1.021, P = 0.059). However, the difference did not reach significance between fibrinolysis and no reperfusion(OR: 0.716, 95% CI:0.441, 1.164, P = 0.178). We found parallel trends of cardiac death and non-cardiac death in the 3 groups. Moreover, when compared with patients in the no reperfusion group, patients in the primary PCI group also had lower rates of heart failure (OR: 0.551, 95% CI: 0.444, 0.683, P < 0.001), mechanic complications (OR: 0.295, 95% CI: 0.106, 0.825, P = 0.02), and cardiac arrest (OR: 0.511, 95% CI: 0.342, 0.763, P = 0.001). However, no difference of that was detected between the fibrinolysis group and the no reperfusion group (P > 0.05).

**Table 4:** The adjusted in-hospital outcomes with different reperfusion strategies.

Variables	Fibrinolysis vs. No reperfusion (OR, 95% CI)	Primary PCI vs. No reperfusion (OR, 95% CI)	Primary PCI vs. Fibrinolysis (OR, 95% CI)
Death	0.716(0.441,1.164)	0.424(0.316,0.569)	0.592(0.343,1.021)
Cardiac death	0.757(0.463,1.238)	0.447(0.331,0.602)	0.590(0.340,1.026)
Non-cardiac Death	0.443(0.058,3.382)	0.310(0.099,0.975)	0.700(0.071,6.870)
Recurrent MI	2.532(0.707,9.065)	0.773(0.291,2.055)	0.305(0.071,1.305)
Heart failure	0.882(0.606,1.283)	0.551(0.444,0.683)	0.624(0.414,0.942)
Mechanic Complications	1.344(0.393,4.593)	0.295(0.106,0.825)	0.220(0.047,1.022)
Cardiac arrest	0.599(0.301,1.193)	0.511(0.342,0.763)	0.853(0.395,1.839)
Cerebral events	1.321(0.389,4.488)	1.218(0.612,2.423)	0.922(0.250,3.393)

The logistic model include variables: GRACE score stratification (categorized as low, moderate high, and very high risk), sex, hospital level, prior stroke, prior heart failure, current smoking, left ventricular ejection fraction, diastolic pressure, use of IABP, in-hospital use of aspirin, ADP inhibitor, LMWH or fondaparinux, statin, β-blocker, ACEI/ARB, nitrate. MI, myocardial infarction

## Discussion

There is very limited evidence in the recommendation of the reperfusion strategy for elderly STEMI patients.[14–20] Age is an independent predictor of adverse outcomes in the elderly, so older patients are in accordance at higher risk of death compared with the younger patients.[21] Immediate reperfusion was proved to be beneficial in younger STEMI patients (< 75 years old) in previous studies. However, older patients were usually excluded, especially in the randomized trials.[22] Older people were more likely to contract MI complications than the younger patients because of the reduced heart function, and the degraded heart structure.[23] Older people were also at higher risk of hemorrhage stroke when they had fibrinolysis, or of gastrointestinal bleeding when they were under dual antiplatelet or triple anticoagulation therapy.[24,25] Older people properly had worse renal function than the younger ones, so the contrast used in primary PCI might elevate the risk of renal failure and contrast-induced nephropathy.[26] Besides, the PCI-related complications such as no-flow, slow flow, vascular dissection or other adverse outcomes could be a heavier blow on the elderly than on the younger patients.[2] Lack of sufficient evidence, it is still unclear if immediate reperfusion was effective and safe in the elderly, or what immediate reperfusion was best, as well. Thus, we used data from a prospective national registry to compare the short outcomes of the older patients with 3 different treatment strategies (primary PCI, fibrinolysis, or no reperfusion).

It is showed that the unadjusted rate of mortality was lower in the primary PCI group than in the fibrinolysis group or in the no reperfusion group. However, after baseline adjustment, this difference was only significant between the primary PCI group and no reperfusion group. This result suggested that primary PCI was an effective reperfusion strategy in the elderly just like in the younger patients, but the efficacy of fibrinolysis in the elderly needs more evidence. The direct comparison of primary PCI and fibrinolysis in older patients was limited. In the real world outcomes, Shelton et al.[14]showed similar rates of 30 d mortality before and after the inception of primary PCI and lower rates of death during a 1- and 3-year follow-up period for primary PCI in older patients, which indicated the benefit of primary PCI in the long run. However, Fosbol et al.[15]found no difference in the long-term mortalities between patients who underwent either primary PCI or fibrinolysis regardless of age. In randomized trials, The SENIOR-PAMI multicenter trial [16]included 130 patients (≥ 80 years old) and showed similar 30 d mortalities between primary PCI and fibrinolysis (PCI 19% vs. Fibrinolysis 16%). The TRIANA trial [17]intermediately terminated because of a non-significant difference in the 30 d mortalities between primary PCI and fibrinolysis in patients ≥ 75 years old. Our result was in consistent with those studies, while the short-term outcomes with primary PCI or fibrinolysis in older patients seemed to be the same. Previous study suggested that prehospital fibrinolysis may be preferable or similar to primary PCI for patients treated within the first 2 hours after symptom onset. So this was also applicable for the older patients.[18]

What unexpected is that we found no significant difference for the short-term mortalities between fibrinolysis and no reperfusion. Previous results were controversial. Thiemann et al.[19]showed that fibrinolysis was harmful for older patients. Older STEMI patients (≥ 75 years old) having fibrinolysis were at higher risk of death at 30 d follow-up compared with patients having no reperfusion (Hazard ratio: 1.38, 95% CI 1.12 to 1.71,P = 0.003). The finding was quite different from the younger patients in the same study, while the mortality rates were 6.8% and 9.8% for the younger patients with or without fibrinolysis. Berger et al.[20] showed similar 30 d mortality between the fibrinolysis group and no reperfusion group in patients ≥ 65 years old, but higher rate of death during 1-year follow-up in the no reperfusion group. The underlying reasons for the inconsistent results between the studies could not be easily interpreted. Firstly, it was thought that older patients were not ideal people for the fibrinolysis strategy, since they were at higher risk of cerebral bleeding, which might compensate the benefit of coronary reperfusion. However, we did not find increased hemorrhage stroke in the fibrinolysis group in this study, which ascertained the safety of fibrinolysis in the elderly. Secondly, even though the success rate of fibrinolysis was high in our study (> 88%), it did not translate into patient's better survival rate. We assumed it could be attributed to the undesirable time from the symptom onset to the beginning of fibrinolysis. Quite a part of patients having fibrinolysis in our study had late fibrionlysis (>12h after the symptom onset, or even >24h), while the effect of late fibrinolysis was diluted after a long period after myocardial infarction. Thirdly, we could not avoid the patient selection bias. In this study, patients having fibrinolysis were of relatively younger age, healthier, and getting more post-fibrinolysis treatments compared with patients having no reperfusion, these disparities could affect and confound the results. So right now, the compare result between fibrinolysis and no reperfusion should be read carefully in the elderly, and more data should be further analyzed.

We found significant difference in the medication used in different groups. Dual antiplatelet therapy, anticoagulation drugs, ACEI/ARB, statin, as well as β-blocker are the first-line drugs that recommended in treating STEMI in the guideline, which could improve the short- and long-term outcomes.[27]Nevertheless, compared with patients in the primary PCI group, these medications were less used in the fibrinolysis group and no reperfusion group. Age was not an contraindication for the antiplatelet drug. However, for the safety concerns, older patients rated as “relatively high bleeding” risk are usually excluded in the primary PCI group because of the concerning of long term dual antiplatelet therapy after stent implantation.[28]GP IIb/IIIa inhibtors was an intravenous antiplatelet drug that recommended in STEMI to improve outcomes. Interventional doctors preferred to perform primary PCI combined with GP IIb/IIIa inhibtors to reduce thrombus. Whereas, the effect of GP IIb/IIIa inhibtors in older patients was still controversial, and the extra bleeding risk related in older patients need to be noticed.[29–31]β-blocker, ACEI/ARB, and statin were recommended to administrate within the first 24 hours after the symptom onset in patients without contraindication.[28]In the “real world” practice, those drugs were underused in the fibrinolysis and no reperfusion group than in the primary PCI group. For all we knew, decisions from the doctors played a leading role in the use of medication. We found the medication used in patients having primary PCI or in the high-level hospitals were more getting with the guideline, but the situation was much worse in patients having no reperfusion or in the tertiary hospitals. Since medication was the footstone for the treatment, doctors were responsible for the prescription regardless of the reperfusion strategy.

### Limitations

This study showed the “realworld” scenario in treating older STEMI patients in China. This analysis was also the first comprehensive investigation specifically focused on patients ≥ 75 years old in China. In this population, we showed and compared the outcomes of patients treated with primary PCI, fibrinolysis, and no immediate reperfusion. However, patients were not randomized, baseline disparities and selection bias was inevitable. A great proportion of late reperfusion in our study may confound the results, 6.9% of the patients having fibrinolysis were between 12 to 24 h, and around 11.3% having fibrinolysis more than 1 d, even though fibrinolysis was usually not intended to be performed>12 h after symptom onset. In addition, drug regimen afterwards with < 50% of patients receiving ACE/ARB and < 55% beta-blockers is also troublesome. However, this showed the real scenario in China. On the other hand, the elderly have not been studied and these data could be interpreted with more caution, but demonstrate a good outcome with primary PCI in this group of patients.

## Conclusions

Elderly patients constitute a significant and increasing proportion of STEMI patients. Early reperfusion, especially primary PCI is safe and effective for elderly patients with absolute reduction of mortality compared with no reperfusion. However, certain randomized trials were encouraged to support the conclusion.
